# A Seven-Rod Dielectric Sensor for Determination of Soil Moisture in Well-Defined Sample Volumes

**DOI:** 10.3390/s19071646

**Published:** 2019-04-06

**Authors:** Justyna Szerement, Aleksandra Woszczyk, Agnieszka Szypłowska, Marcin Kafarski, Arkadiusz Lewandowski, Andrzej Wilczek, Wojciech Skierucha

**Affiliations:** 1 Institute of Agrophysics, Polish Academy of Sciences, Doświadczalna 4, 20-290 Lublin, Poland; a.woszczyk@ipan.lublin.pl (A.W.); a.szyplowska@ipan.lublin.pl (A.S.); m.kafarski@ipan.lublin.pl (M.K.); a.wilczek@ipan.lublin.pl (A.W.); w.skierucha@ipan.lublin.pl (W.S.); 2 Institute of Electronic Systems, Warsaw University of Technology, Nowowiejska 15/19, 00-665 Warsaw, Poland; a.lewandowski@elka.pw.edu.pl

**Keywords:** FDR, TDR, dielectric permittivity, soil moisture, dielectric spectroscopy

## Abstract

This paper presents a novel seven-rod sensor used for time-domain reflectometry (TDR) and frequency-domain reflectometry (FDR) measurements of soil water content in a well-defined sample volume. The probe directly measures the complex dielectric permittivity spectrum and for this purpose requires three calibration media: air, water, and ethanol. Firstly, electromagnetic simulations were used to study the influence of the diameter of a container on the sensitivity zone of the probe with respect to the measured calibration media and isopropanol as a verification liquid. Next, the probe was tested in three soils—sandy loam and two silt loams—with six water contents from air-dry to saturation. The conversion from $S_{11}$ parameters to complex dielectric permittivity from vector network analyzer (VNA) measurements was obtained using an open-ended liquid procedure. The simulation and measurement results for the real part of the isopropanol dielectric permittivity obtained from four containers with different diameters were in good agreement with literature data up to 200 MHz. The real part of the dielectric permittivity was extracted and related to the moisture of the tested soil samples. Relations between the volumetric water content and the real part of the dielectric permittivity (by FDR) and apparent dielectric permittivity (by TDR) were compared with Topp’s equation. It was concluded that the best fit to Topp’s equation was observed in the case of a sandy loam. Data calculated according to the equation proposed by Malicki, Plagge, and Roth gave results closer to Topp’s calibration. The obtained results indicated that the seven-rod probe can be used to accurately measure of the dielectric permittivity spectrum in a well-defined sample volume of about 8 cm^3^ in the frequency range from 20 MHz to 200 MHz.

## 1. Introduction

There is a pressing need for an indirect, rapid, and accurate moisture content measurement method in porous media [1]. The direct laboratory methods currently used are often expensive, time-consuming, destructive, and labor-intensive [2]. Precise irrigation scheduling is not possible using direct methods in some cases; for example, when trying to apply laboratory methods to measure moisture in a well-defined small volume (inter alia in soil moisture observation conducted near the root system) [3,4]. The development of dielectric measurement techniques in the time and frequency domain in a broad frequency range became very useful for the fast and nondestructive in situ determination of the soil volumetric water content (θ) [5].

Dielectric techniques rely on the determination of the relationship between dielectric permittivity and soil water content. Dielectric permittivity is a complex number $\varepsilon^{*}\left(f\right)=\varepsilon^{\prime }\left(f\right)-j\varepsilon^{\prime \prime }\left(f\right)$, with real ($\varepsilon^{\prime }$) and imaginary ($\varepsilon^{\prime \prime }$) parts that exhibit frequency f dependence. As the relative dielectric permittivity of free water is much higher than the dielectric permittivity of other soil components, soil water content is the main factor influencing the permittivity, especially its real part [6].

The application of time-domain reflectometry (TDR) to determine soil water content is becoming the most commonly used technique. The main advantages of TDR over other methods of determination of θ are as follows: superior accuracy within 1% or 2% volumetric water content; calibration requirements are minimal; lack of radiation hazard associated with the neutron probe or gamma ray attenuation techniques; and the TDR technique can provide excellent spatial and temporal resolution [7,8,9]. TDR gives an averaged value of apparent permittivity ($\varepsilon_{a}$) which is composed of both $\varepsilon^{\prime }$ and $\varepsilon^{\prime \prime }$. The first TDR calibration was proposed by Topp et al. [10] in 1980. This universal calibration has been used in numerous reports for years up to now and is regarded as a reference for data obtained using different sensors. The main drawbacks of this method are that the TDR sensors may not correctly estimate the water content in soils with relatively high salinity and the measurement equipment is generally very expensive [11].

The frequency-domain reflectometry (FDR) technique represents an alternative to TDR, providing relatively inexpensive measurement of soil water content. This technique enables measuring the $\varepsilon^{\prime }$ and $\varepsilon^{\prime \prime }$ parts of the complex dielectric permittivity of soil. In order to use FDR sensors to determine soil moisture, one needs to apply a $\theta -\varepsilon^{\prime }$ calibration function valid for the $\varepsilon^{\prime }$ value obtained at the operational frequency of a given sensor [12].

Typically, the capacitance sensors based on FDR operate at a single frequency in the range of 5–100 MHz [13]. Several polarization effects can be observed in those frequencies, including double-layer effects. Measurements above 100 MHz minimize the effect of Maxwell–Wagner polarization [14]. On the other hand, higher clay content causes dispersion up to 1 GHz [15,16]. For this reason, the development of sensors operating in a broadband frequency range including frequencies higher than 100 MHz is advantageous for the purpose of minimizing the influence of clay content on the accuracy of soil moisture determination.

Study of the dielectric permittivity spectrum can provide valuable information about the polarization effects occurring with increasing frequency. There are a few papers available on subjects that deal with the development of sensors based on FDR measuring of the dielectric spectrum of permittivity in frequencies above 100 MHz. In the literature, the two-rod FDR sensor and measurement methodology of $\varepsilon^{\prime }$ in the 10–500 MHz was discussed [12]. It was discovered that the measurement of soil moisture in the frequency range 390–480 MHz was comparable with that by TDR. Another paper also proposed the utilization of FDR sensors in measuring soil water content in the frequency range of 47–200 MHz [17].

The accuracy and performance of the sensors not only depend on frequency and soil properties but are also related to their geometry. Soil moisture sensors should have good contact with the soil material without any gaps between the sensing element and the surface of the material. Some papers discussed soil water content determination using an open-ended probe and an open-ended probe with an antenna [18]. However, this probe’s sensitivity zone was too small to characterize highly heterogeneous material such as soil [19]. There has also been the development of a sensor consisting of two or more rods encircling a signal one [20]. In this sensor, the number and length of the rods determine the geometry of the electrical field distribution with minimized field leakage outside of the sensor. Electromagnetic simulations of a short thermo-FDR consisting of two, three, four, and six outside needles were proposed in reference [17]. The authors indicated that the electric field distribution of the sensor with six outer needles was similar to that of a coaxial transmission line. In this paper, the measured $S_{11}$ parameter was used to extract the permittivity according to the impedance procedure described in reference [21].

The aim of the study was to construct, electromagnetically simulate, and test a seven-rod probe of a simplified coaxial geometry consisting of a signal rod surrounded by six ground ones. The purpose of choosing such a geometry was to provide a well-defined sensitivity zone that is large enough for an accurate measurement of a granular material, yet small enough to be practical for laboratory applications. Because this paper concentrates on soil moisture determination, only $\varepsilon^{\prime }$ spectra are presented.

This paper presents the following:

- electromagnetic simulations used to verify the sensitivity zone of the probe using Ansys HFSS software;
- measurements of the $\varepsilon^{\prime }$ values of three soils of different texture and moisture content using the seven-rod probe connected to a vector network analyzer (VNA) in the determined operational frequency range of the probe;
- conversion of $S_{11}$ parameters obtained from VNA measurements to complex permittivity using a bilinear equation (Open-Water-Liquid calibration);
- transient-state electronic simulations in the Keysight Advanced Design System (ADS), which enabled converting the $S_{11}$ parameters measured by the VNA from the frequency domain to the time domain in order to obtain $\varepsilon_{a}$ and to verify that the probe could be used also as a TDR probe;
- a comparison between $\varepsilon^{\prime }$, $\varepsilon_{a}$, and volumetric moisture content from the experiment;
- determination of the permittivity–moisture content calibration curve and its comparison to Topp’s equation as reference.

## 2. Materials and Methods

### 2.1. Sensitivity Zone—Simulation and Experiment

**Figure 1:**
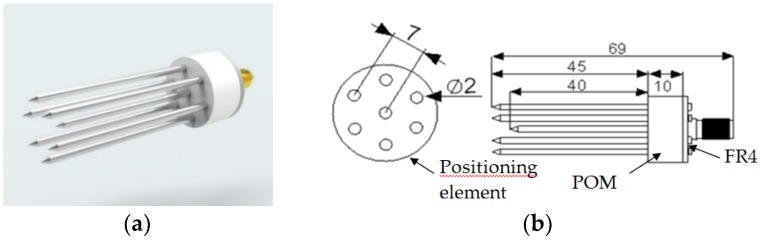
Tested seven-rod probe and the elements of the measurement setup; (**a**) a probe; (**b**) respective dimensions of the probe (dimensions in mm).

In this study, dielectric properties were measured using a seven-rod probe manufactured at the Laboratory of Dielectric Spectroscopy of the Institute of Agrophysics PAS, Lublin, Poland (Figure 1a). The sensor is equipped with seven rods made of acid-proof steel. The signal rod is surrounded by six ground rods. The rods and SMA socket were soldered to the round plate of the Printed Circuit Board (PCB) composed primarily of FR4 circuit board material. The positioning element was made of poliacetal (POM). The dimensions of the probe are shown in Figure 1b.

**Figure 2:**
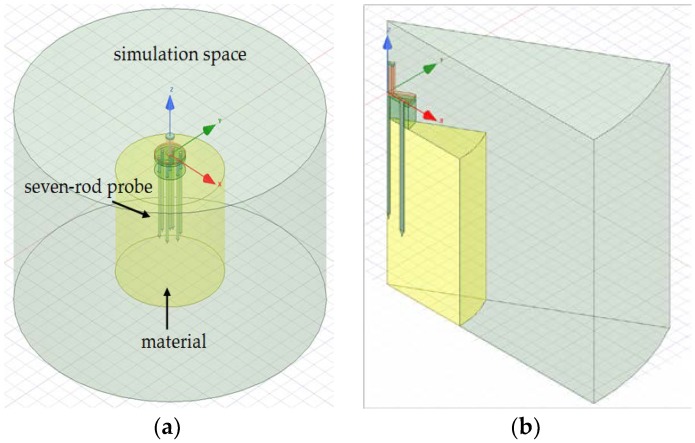
The model of (**a**) the whole simulation space; (**b**) 30 degree sector cut from the whole simulation space consisting of a signal rod and ground ones (dimensions in mm).

Simulations of the seven-rod probe were performed using the Ansys HFSS software pack, which uses a finite element method (FEM) for calculation [22]. The model of the simulation space is presented in Figure 2a,b.

The dimensions of the probe used in the simulations were identical to those of the probe which is shown in Figure 1. The probe was surrounded by material in the form of a cylinder 70 mm in height and 70, 35, 20, or 15 mm in diameter (*ϕ*) filled with calibration media (air, water, and ethanol) and isopropanol as a verification liquid.

In Figure 2a, a full model of the probe used in simulations is shown. Since the structure is periodic around the vertical axis, we used only a 30 degree sector cut from the original structure and put magnetic walls on both cutting surfaces to enforce the original field symmetry; the resulting structure is shown in Figure 2b. In this way it was possible to significantly reduce the memory usage of the EM simulator and speed up the simulation process. All other boundaries were set to be absorbing conditions.

The experiment was carried out using the probe prototype connected to a one-port VNA R60 (Cooper Mountain Technologies) using the compatible SMA/Type-N adapter. The probe was inserted into containers with the same geometry filled with air, water, ethanol, and isopropanol. Respective $S_{11}$ parameters were simulated and measured in a frequency range from 1 MHz to 6 GHz, and the number of frequency points was 325 (50, 195, and 80 points in the intervals 1 MHz–50 MHz, 60 MHz–2 GHz, and 2.05 GHz–6 GHz, respectively).

Because the seven-rod probe has a simplified coaxial geometry, open-water-liquid (OWL) calibration was chosen. The conversion from $S_{11}$ parameters to complex permittivity $\left(\varepsilon^{*}\right)$ was obtained using the following bilinear equation:

(1) $$\varepsilon^{*}=\frac{c_{1}S_{11}-c_{2}}{c_{3}-S_{11}}$$

where the calibration constants $c_{1},c_{2}$, and $c_{3}$ are complex numbers determined in the OWL calibration using air, distilled water, and ethanol as the calibration media [23,24]. The obtained $\varepsilon^{\prime }$ value of isopropanol (from the simulation and the experiment) was compared with the data found in available literature [25].

### 2.2. Soil Characteristics and Measurements

The following three soils of various textures were examined: silt loam (GRT) and two sandy loams (SKR and ZLZ). Soil samples were collected from a depth of 20–30 cm below the surface and sieved using a 2 mm sieve. The texture and surface area data are presented in Table 1. The clay, silt, and sand contents were determined from the particle size distribution measured using a laser diffraction method according to reference [26]. Determination of the specific surface area was based on the Brunauer–Emmett–Teller (BET) gas adsorption method using nitrogen.

The experimental setup consisted of the seven-rod probe prototype connected to the VNA and a plastic container 70 mm in *ϕ* and 70 mm in height. All measurements were conducted on each of the soils with six water contents covering a range of water contents from air-dry soil to soil saturation. After the water was added, the soil was thoroughly mixed, covered to avoid evaporation, and allowed to stand overnight at 20 ± 1 °C to obtain a homogeneous sample before measurements were taken. The highest saturation points and specific surface areas for the GRT and ZLZ soils were similar; however, they differed in clay content. The value of specific surface area depends on the content and mineral composition of the clay fraction and on the content of organic matter and amorphous oxides [27]. This indicates that it is not only the clay content that contributes to the rise of the specific surface area in ZLZ soil. In the case of SKR soil, its specific surface area was about 5 times lower compared to those of the remaining soils. The seven-rod probe was carefully inserted into the soil to ensure proper contact between the probe rods and the surrounding soil. Following measurement, the θ value was determined using a thermogravimetric method according to PN-ISO 11465:1999—Polish version [28]. Because all tested soil samples were air-dry at the start of the experiment, an initial water content was included in the calculations. The experiment was conducted at 20 ± 1 °C in three independent replications.

**Figure 3:**
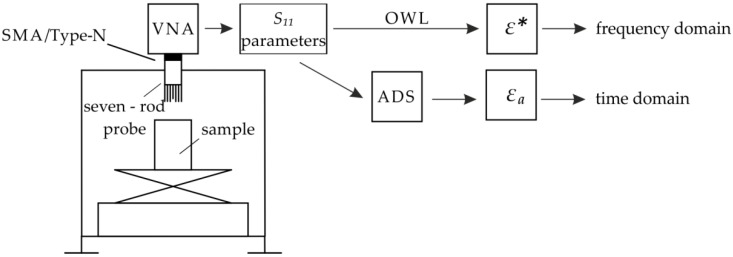
Overview of results processing.

The $S_{11}$ parameters obtained from VNA were used for conversion to $\varepsilon^{*}$ using the OWL procedure described earlier. These parameters were also imported to the ADS software for further processing in the time domain with the use of transient-state analysis [29]. An overview of the results processing is shown in Figure 3.

**Figure 4:**
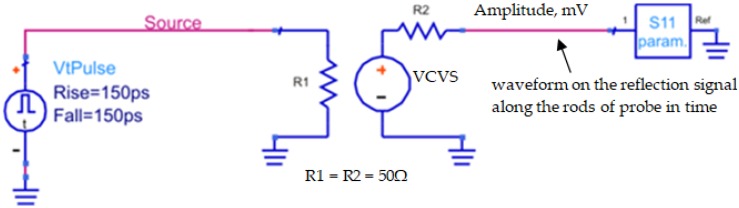
The scheme of the ADS transformation from the frequency domain to the time domain [31].

The elementary electric circuit used in the ADS [30] is presented in Figure 4, where the VtPulse is a needle pulse generator with 150 ps rise and fall times and 1 V amplitude; $S_{11}$ stands for the element of the scattering matrix imported from the VNA; and R1, R2, and the Voltage Controlled Voltage Source (VCVS) match and separate the signal path between the needle pulse generator and the probe represented by $S_{11}$.

The ADS analysis provided the reflectograms showing the time and amplitude of reflections from the seven-rod probe as a response to the initial needle pulse of 150 ps. From the obtained reflection times, the $\varepsilon_{a}$ value of each sample was determined.

## 3. Results and Discussion

### 3.1. Frequency Domain Analysis

**Figure 5:**
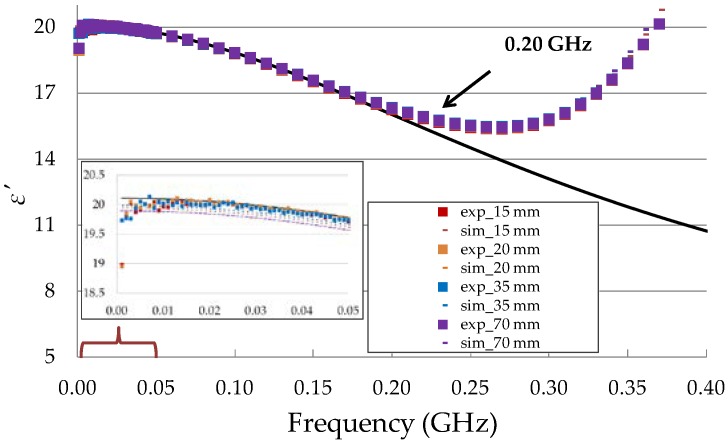
Comparison of $\varepsilon^{\prime }$ values for isopropanol obtained from simulations (sim) and experiments (exp) for measurements in containers with different diameters (in mm). The black solid line corresponds to the Debye spectrum of isopropanol data obtained from available literature (T = 20 °C, $\varepsilon_{s}$ = 29, $\varepsilon_{\infty }$ = 3.2, τ = 292 ps) [25].

The simulation and experimental measurement data of the $\varepsilon^{\prime }$ values of isopropanol obtained from four containers with different *ϕ* were in good agreement with the literature data [25] up to 200 MHz (Figure 5). The low values of root-mean-square error (RMSE) between the measured and simulated $\varepsilon^{\prime }$ values and the known reference permittivity of isopropanol indicated that the *ϕ* of the container did not affect the measurement of the $\varepsilon^{\prime }$ value of isopropanol (Table 2). Hence, it can be concluded that the sensitivity zone of the seven-rod probe corresponded to the small volume between the six ground conductors of about 8 cm^3^. The conversion of the $S_{11}$ parameter to dielectric permittivity in the FDR method gave correct data in the frequency range from 20 MHz to 200 MHz, as illustrated in Figure 5. The scattering of the ε′ values of the tested FDR sensor prototype increased at frequencies below 20 MHz. Other studies [18,23] reported an increase in measurement error at low frequencies for an open-ended probe calibrated using the same OWL procedure. The limit of the working frequency (200 MHz) for the tested FDR sensor can be explained by the resonance effect. A high-frequency limit to the applicability of an OWL calibration procedure was also observed for an open-ended probe with an antenna [18,23].

**Figure 6:**
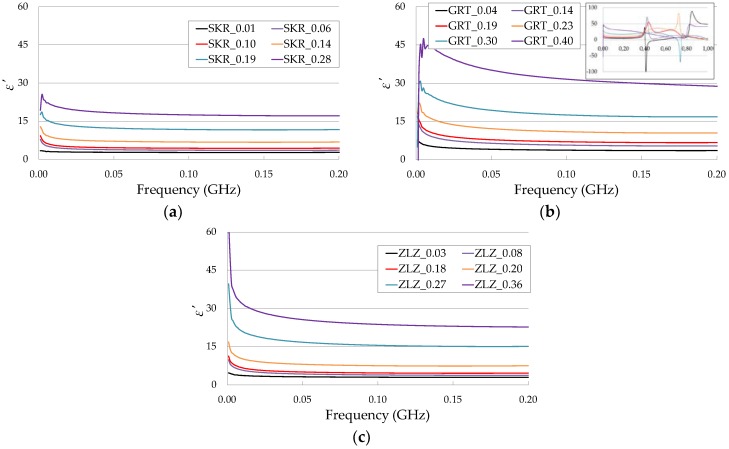
The $\varepsilon^{\prime }$ values of SKR (**a**), GRT (**b**), and ZLZ (**c**) soil samples with different water contents over the frequency range from 1 MHz to 200 MHz. The name of each sample consists of the type of soil and the θ value. Three VNA measurements from various soil samples were performed for each moisture content. The curves present the average values with the maximum value of the standard error ± 3%.

The sample $\varepsilon^{\prime }$ spectra obtained using the seven-rod probe over the frequency range from 1 MHz to 200 MHz are presented in Figure 6. The value of $\varepsilon^{\prime }$ in each of the samples increased with increasing water content, which is consistent with other studies [32]. The limit to the working frequency for the tested probe at low frequencies for analyzed soils was caused by low-frequency interfacial polarization effects.

It was also observed that increasing frequency caused a reduction in the $\varepsilon^{\prime }$ value. A strong increase of $\varepsilon^{\prime }$ was mainly noted at low frequencies, especially in GRT and ZLZ soils containing greater clay and silt fractions. This could be explained by the interfacial polarization effects connected with the presence of clay particles [33].

**Figure 7:**
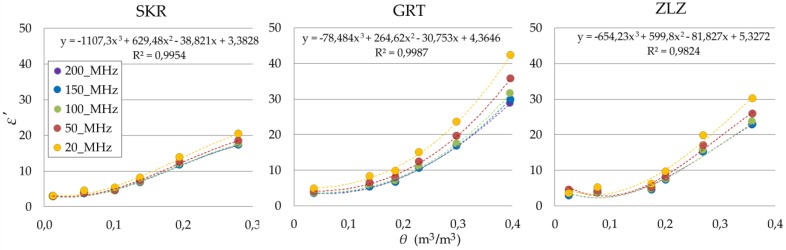
Relation between θ and ε′ of the tested soils (SKR, GRT, ZLZ) at 20 MHz, 50 MHz, 100 MHz, 150 MHz and 200 MHz. Dashed lines represent third degree polynomials fitted to the data. Equations refer to data at 200 MHz.

The relations between the θ and $\varepsilon^{\prime }$ values of the tested soils at a chosen frequency are presented in Figure 7. Third-degree polynomials were fitted to the data in order to obtain soil moisture–dielectric permittivity calibration curves.

In the case of the SKR soil, the increasing frequency did not change the ε′, except for the saturated sample. However, in other soils that contained more clay fraction, the ε′ generally differed with frequency, especially at higher θ. The results obtained in relation to GRT and ZLZ soils could be explained by strong water–clay interactions in low frequencies. The third degree polynomial fitted to obtained data for 200 MHz shows high $R^{2}$ for tested soils.

### 3.2. Time Domain Analysis

**Figure 8:**
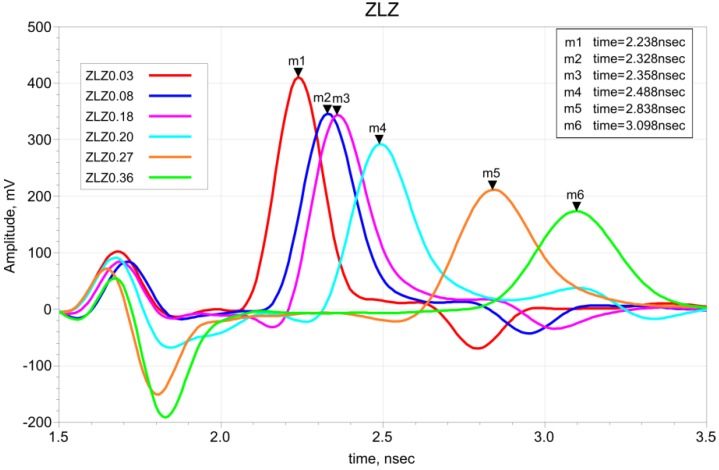
Examples of waveforms in the time domain from ADS for ZLZ soil samples with different θ values.

The $S_{11}$ parameters obtained from VNA measurements were used for travel time calculations using the ADS software. Examples of waveforms for different moisture contents of ZLZ soil are presented in Figure 8. The first negative reflection comes from the beginning of the probe rods and the positive second one is the reflection from the ends of the probe rods. For each sample, the travel time of the positive second reflection is presented in Figure 8.

**Figure 9:**
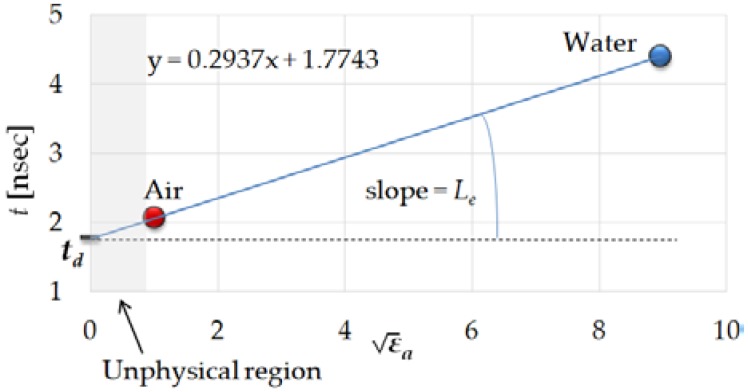
Relations between t values of water and air calculated from ADS software for those calibration media and $\sqrt{\varepsilon_{a}}$.

The delay time (*t_d_*) and electrical length (*L_e_*) of the internal signal rod at the probe must be calculated, according to reference [34]. The solution of the appropriate system of equations can be graphically illustrated as shown in Figure 9.

**Figure 10:**
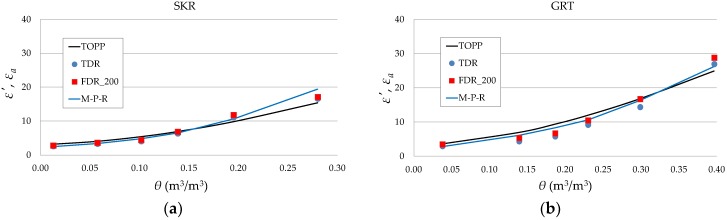

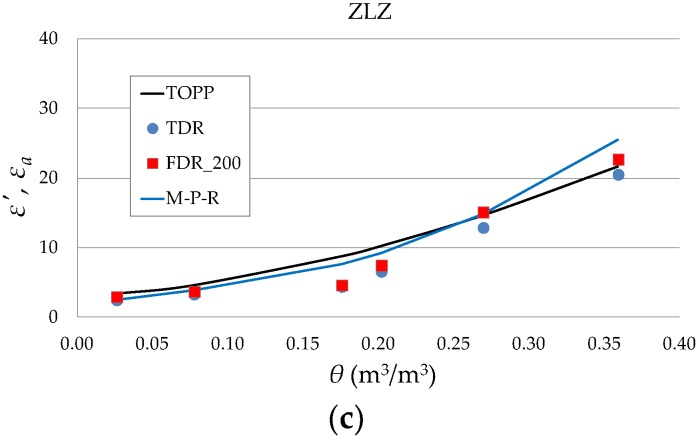
Relations between $\varepsilon^{\prime }$ values at 200 MHz. The $\varepsilon_{a}$ (data from ADS simulation and the Malicki–Plagge–Roth (M-P-R) equation) and θ values of tested soils (data from measurements using the seven-rod probe and ADS simulations). Black solid lines refer to Topp’s equation and blue solid lines illustrate the M-P-R equation with density correction (Equation (2)).

The relations between $\varepsilon^{\prime }$, $\varepsilon_{a}$, and θ of the tested soils are shown in Figure 10. Because the measured samples differed in density (Table 3), the $\varepsilon_{a}$ value was calculated according to the formula presented in reference [35], the Malicki–Plagge–Roth equation (M-P-R), which included a correction for soil density (ρ):

(2) $$\sqrt{\varepsilon^{*}}\;\left(\theta ,\rho \right)=0.819+0.168\rho +0.159\rho^{2}+\left(7.17+1.18\rho \right)\theta .$$

Topp’s relation for $\varepsilon_{a}$ [10],

(3) $$\varepsilon_{a}=3.03+9.3\theta +146.0\theta^{2}-76.7\theta^{3},$$

is also presented in Figure 10 for the purpose of comparison with the obtained results (black solid lines).

In general, Topp’s equation overestimated the values of $\varepsilon^{\prime }$ and $\varepsilon_{a}$ for GRT and ZLZ soils, except for air-dry and saturated soils. In SKR soil the values of $\varepsilon^{\prime }$ were in line with the values from Topp’s equation and TDR up to θ = 0.14 m^3^/m^3^. As noted in the available literature. the equation proposed by Topp [10] does not fit well in the case of fine-grained soils [36] and any soils with high organic matter content [37]. This may occur due to the increase in the specific surface area of the soil with the increase in clay content, which is the reason for significant influence from the adsorbed layer of water. Hence, there are ongoing efforts to develop equations that better describe the relations between dielectric permittivity and soil water content by taking into account their physical properties. The calibration proposed by M-P-R takes into account the density of the material in calculations of permittivity. As can be seen in Figure 10, the applied density correction provided higher values of dielectric permittivity for GRT and ZLZ soils which were closer to those from Topp’s equation.

Lower values of permittivity were observed in the data obtained from ADS (TDR) in each of the soil samples. Conversion of the data from $S_{11}$ parameters to the time domain was taken from the whole measured frequency range of the probe (from 1 MHz to 6 GHz), unlike in the case of FDR where the range is only up to 200 MHz. Time domain $\varepsilon_{a}$ data calculated from the ADS represent higher-frequency values of $\varepsilon^{\prime }$ which are smaller than those for 200 MHz. Additionally. it is possible that electrical field leaks out of the zone between the sensor rods at high frequencies. This will be investigated in a future study.

## 4. Conclusions

The obtained data from electromagnetic simulations and measurements indicated that the geometry of the proposed seven-rod sensor enabled us to measure soil moisture in a well-defined volume of 8 cm^3^ in the frequency range of 20–200 MHz. The operational frequency range of the tested probe can be explained by limitations in the OWL calibration procedure. The probe was tested in soil differing in texture with six water contents from air-dry to saturation. The correlation between $\varepsilon^{\prime }$ at 200 MHz gave high values of R^2^ for the tested soils, especially for SKR soil. Generally, the $\varepsilon^{\prime }$ values measured by VNA were in good agreement with the $\varepsilon_{a}$ values calculated from $S_{11}$ parameters determined by the TDR method and obtained from ADS software. In the case of SKR, the values of $\varepsilon^{\prime }$ and $\varepsilon_{a}$ agreed well when fit with Topp’s equation. For GRT and ZLZ soils containing greater clay and silt fractions, it was concluded that the Topp’s values were overestimated, except for air-dry and saturated soils. The application of the seven-rod FDR sensor to measuring the water content of samples in a well-defined, small volume with high accuracy may be useful in laboratory research, e.g., in soil moisture monitoring near the root system and for precise irrigation scheduling. Furthermore, the measurements obtained from the seven-rod sensor may be useful in the development of a standard calibration methodology for other sensors operating within the 20–200 MHz frequency range. Some planned future work includes the development of other conversion formulas $\varepsilon^{*}=f\left(S_{11}\right)$ for the presented sensor in order to extend its operational frequency range.

## Figures and Tables

**Table 1:** Soil characteristics of silt loam (GRT) and two sandy loams (SKR and ZLZ).

	Texture [%]			Specific Surface Area [m^2^/g]
	Clay	Silt	Sand	
SKR	3.7	28.8	67.5	3.06 ± 0.06
GRT	10.1	76.5	13.4	16.40 ± 0.02
ZLZ	5.1	44.2	50.7	15.41 ± 0.30

**Table 2:** Root-mean-square error (RMSE) between the measured and simulated $\varepsilon^{\prime }$ values for measurements in containers with different diameters (in mm) and the accepted value for isopropanol in the literature [25].

*ϕ*	Simulation	Experiment
70 mm	0.042	0.023
35 mm	0.016	0.012
20 mm	0.014	0.026
15 mm	0.025	0.027

**Table 3:** Density of samples.

SKR		GRT		ZLZ	
*θ* [m^3^/m^3^]	*ρ* [g/cm^3^]	*θ* [m^3^/m^3^]	*ρ* [g/cm^3^]	*θ* [m^3^/m^3^]	*ρ* [g/cm^3^]
0.01	1.54	0.04	1.35	0.03	1.38
0.06	1.36	0.14	1.24	0.08	1.29
0.10	1.31	0.19	1.25	0.18	1.21
0.14	1.36	0.23	1.25	0.20	1.27
0.19	1.60	0.30	1.44	0.27	1.51
0.28	1.96	0.40	1.63	0.36	1.86
